# Benzyl 2-methyl-3-[(*E*)-(thio­phen-2-yl)methyl­idene]dithio­carbazate

**DOI:** 10.1107/S1600536812012652

**Published:** 2012-03-28

**Authors:** Saroj K. S. Hazari, B. K. Dey, Tapashi G. Roy, B. Ganguly, Seik Weng Ng, Edward R. T. Tiekink

**Affiliations:** a Department of Chemistry, University of Chittagong, Chittagong 4331, Bangladesh; b Department of Chemistry, University of Malaya, 50603 Kuala Lumpur, Malaysia; c Chemistry Department, Faculty of Science, King Abdulaziz University, PO Box 80203 Jeddah, Saudi Arabia

## Abstract

In the title compound, C_14_H_14_N_2_S_3_, the thione S atom and methyl group are *syn*, as are the two thio­ether S atoms. The mol­ecule is twisted, the dihedral angles between the central (C_2_N_2_S_2_) residue and the pendent 2-thienyl and phenyl rings being 21.57 (6) and 77.54 (3)°, respectively. In the crystal, mol­ecules assemble into a three-dimensional architecture *via* C—H⋯π inter­actions, involving both the five- and six-membered rings as acceptors, as well as S⋯S inter­actions [3.3406 (5) Å] between centrosymmetrically related 2-thienyl rings.

## Related literature

For the biological activity of related Schiff base compounds, see: Hazari *et al.* (2002 ▶). For a related structure, see: Scovill & Silverton (1980 ▶).
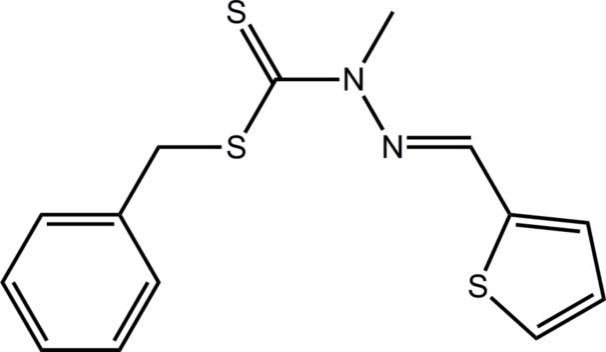


## Experimental

### 

#### Crystal data

C_14_H_14_N_2_S_3_
*M* *_r_* = 306.45
Monoclinic, 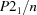

*a* = 6.0585 (1) Å
*b* = 19.3774 (5) Å
*c* = 12.5769 (3) Å
β = 103.200 (2)°
*V* = 1437.49 (6) Å^3^
*Z* = 4
Cu *K*α radiation
μ = 4.60 mm^−1^
*T* = 100 K
0.15 × 0.15 × 0.15 mm

#### Data collection

Agilent SuperNova Dual diffractometer with an Atlas detector
Absorption correction: multi-scan (*CrysAlis PRO*; Agilent, 2011 ▶) *T*_min_ = 0.871, *T*_max_ = 1.000
5742 measured reflections
2958 independent reflections
2746 reflections with *I* > 2σ(*I*)
*R*_int_ = 0.017

#### Refinement

*R*[*F* ^2^ > 2σ(*F* ^2^)] = 0.029
*wR*(*F* ^2^) = 0.080
*S* = 1.04
2958 reflections
173 parameters
H-atom parameters constrained
Δρ_max_ = 0.29 e Å^−3^
Δρ_min_ = −0.50 e Å^−3^

### 

Data collection: *CrysAlis PRO* (Agilent, 2011 ▶); cell refinement: *CrysAlis PRO*; data reduction: *CrysAlis PRO*; program(s) used to solve structure: *SHELXS97* (Sheldrick, 2008 ▶); program(s) used to refine structure: *SHELXL97* (Sheldrick, 2008 ▶); molecular graphics: *ORTEP-3* (Farrugia, 1997 ▶) and *DIAMOND* (Brandenburg, 2006 ▶); software used to prepare material for publication: *publCIF* (Westrip, 2010 ▶).

## Supplementary Material

Crystal structure: contains datablock(s) global, I. DOI: 10.1107/S1600536812012652/hg5198sup1.cif

Structure factors: contains datablock(s) I. DOI: 10.1107/S1600536812012652/hg5198Isup2.hkl

Supplementary material file. DOI: 10.1107/S1600536812012652/hg5198Isup3.cml

Additional supplementary materials:  crystallographic information; 3D view; checkCIF report

## Figures and Tables

**Table 1:** Hydrogen-bond geometry (Å, °) *Cg*1 and *Cg*2 are the centroids of the S1,C1–C4 and C9–C14 rings, respectively.

*D*—H⋯*A*	*D*—H	H⋯*A*	*D*⋯*A*	*D*—H⋯*A*
C6—H6*A*⋯*Cg*1^i^	0.98	2.85	3.4021 (17)	117
C8—H8*A*⋯*Cg*2^ii^	0.99	2.80	3.4795 (15)	127

Symmetry codes: (i) 
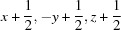
; (ii) 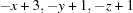.

**Fig. 1.**
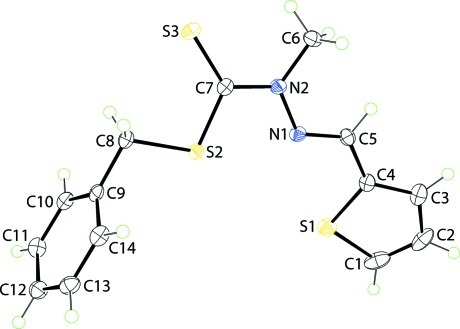
The molecular structure of (I) showing the atom-labelling scheme and displacement ellipsoids at the 50% probability level.

**Fig. 2.**
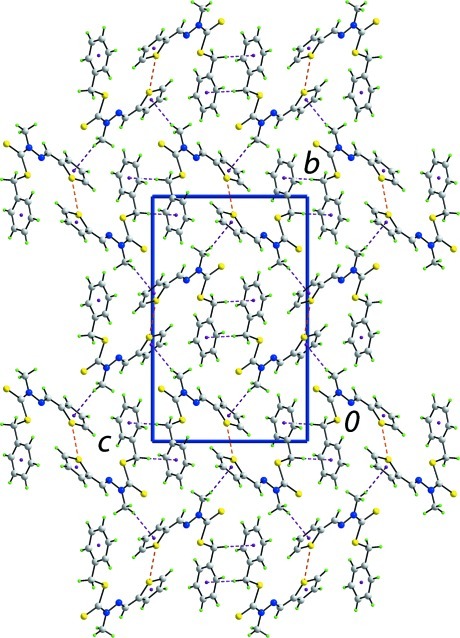
A view of the unit-cell contents in projection down the a axis in (I). The C—H···π and S···S interactions are shown as purple and orange dashed lines, respectively.

## supplementary crystallographic information

### Comment

As a continuation of systematic studies into the synthesis, characterization and biological activities of substituted Schiff base ligands and their metal complexes (Hazari *et al.*, 2002), crystals of the title compound, (I), were isolated and characterized crystallographically.

In (I), Fig. 1, the conformation about the imine N1═C5 bond [1.2876 (18) Å] is *E*. The six atoms of the central residue (S2,S3,N1,N2,C6 & C7) are co-planar having a r.m.s. deviation for the fitted atoms of 0.0395 Å. The maximum deviations from this plane are 0.0478 (7) Å for the S2 atom and -0.0557 (7) Å for the N1 atom. The 2-thienyl and phenyl rings form dihedral angles of 21.57 (6) and 77.54 (3)°, respectively, with the central plane, indicating a twisted molecule. In a rare example of a closely related compound, an *S*-methyl ester, the extreme ends of the molecule are both co-planar with the central residue (Scovill & Silverton, 1980). In (I), the thione-S and methyl group are *syn*, as are the two thioether-S atoms.

Molecules are consolidated into a three-dimensional architecture by C—H···π interactions, involving both the five- and six-membered rings as acceptors, Table 1, as well as S1···S1^i^ interactions [distance = 3.3406 (5) Å for symmetry operation *i*: 1 - *x*, 1 - *y*, -*z*] between centrosymmetrically related 2-thienyl rings, Fig. 2.

### Experimental

The title compound was isolated after a four step synthetic procedure. Synthesis of *N*-methyl-*S*-benzyldithiocarbazate: Potassium hydroxide (11.5 g), was dissolved in 90% ethanol (60 ml) and the mixture was cooled down to 273 K in an ice-bath. To this, methyl hydrazine (11.1 ml) was added slowly with mechanical stirring. A solution of carbondisulfide (12 ml) was added drop-wise from a burette with constant stirring over a period of 1 h. During this addition, the temperature of the reaction mixture was not to allowed to rise above 279 K. A yellow solution was obtained. Benzyl chloride (25 mL) was then added drop-wise with vigorous mechanical stirring. After the complete addition, the mixture was stirred for further 15 min, whereupon well formed crystals appeared. The product was separated by filtration and washed with water and recrystallized from ethanol and dried in a vacuum desiccator over silica gel. Yield: 15.75 g. *M*.pt: 373–375 K.

Synthesis of (I): A hot solution thiophene-2-carboxaldehyde (1.05 ml, 10 mmol) in absolute ethanol (40 ml) was mixed with a hot solution of *N*-methyl-*S*-benzyldithiocarbazate (2.12 g, 10 mmol) in the same solvent (40 ml). The mixture was refluxed for 6 h. on a water bath. After reducing a pale-red product appeared which was filtered off. This product was washed with ethanol several times (3 × 2 ml) and dried in a vacuum desiccator over silica gel. Yield: 1.55 g. *M*.pt: 433–435 K.

Attempted preparation of the dioxomolybdenum(VI) complex with (I): [MoO_2_(acac)_2_] (10 mmol) was dissolved in dry ethanol (40 ml) to which a hot solution of *L* (10 mmol) in dry ethanol (40 ml) was added. The mixture was refluxed for 6 h. on a water bath. After reducing the volume and standing overnight a light-blue product appeared, which was washed with ethanol for several times and dried in a vacuum desiccator over silica gel. *M*. pt: of product was > 493 K. Crystallization: The product was dissolved in ethanol to which half volume of petroleum ether was added (10/5 ml *v*/*v*). The solution was left for several days after which the title compound, (I), was deposited as crystals.

### Refinement

The C-bound H-atoms were placed in calculated positions (C—H = 0.95–0.99 Å) and were included in the refinement in the riding model approximation, with *U*_iso_(H) = 1.2–1.5*U*_equiv_(C).

### Crystal data

**Table:**

C_14_H_14_N_2_S_3_	*F*(000) = 640
*M**_r_* = 306.45	*D*_x_ = 1.416 Mg m^−^^3^
Monoclinic, *P*2_1_/*n*	Cu *K*α radiation, λ = 1.54184 Å
Hall symbol: -P 2yn	Cell parameters from 3411 reflections
*a* = 6.0585 (1) Å	θ = 3.6–76.3°
*b* = 19.3774 (5) Å	µ = 4.60 mm^−^^1^
*c* = 12.5769 (3) Å	*T* = 100 K
β = 103.200 (2)°	Block, yellow-green
*V* = 1437.49 (6) Å^3^	0.15 × 0.15 × 0.15 mm
*Z* = 4	

### Data collection

**Table:**

Agilent SuperNova Dual diffractometer with an Atlas detector	2958 independent reflections
Radiation source: SuperNova (Cu) X-ray Source	2746 reflections with *I* > 2σ(*I*)
Mirror monochromator	*R*_int_ = 0.017
Detector resolution: 10.4041 pixels mm^-1^	θ_max_ = 76.5°, θ_min_ = 4.3°
ω scan	*h* = −7→6
Absorption correction: multi-scan (*CrysAlis PRO*; Agilent, 2011)	*k* = −21→24
*T*_min_ = 0.871, *T*_max_ = 1.000	*l* = −14→15
5742 measured reflections	

### Refinement

**Table:**

Refinement on *F*^2^	Primary atom site location: structure-invariant direct methods
Least-squares matrix: full	Secondary atom site location: difference Fourier map
*R*[*F*^2^ > 2σ(*F*^2^)] = 0.029	Hydrogen site location: inferred from neighbouring sites
*wR*(*F*^2^) = 0.080	H-atom parameters constrained
*S* = 1.04	*w* = 1/[σ^2^(*F*_o_^2^) + (0.0498*P*)^2^ + 0.373*P*] where *P* = (*F*_o_^2^ + 2*F*_c_^2^)/3
2958 reflections	(Δ/σ)_max_ = 0.001
173 parameters	Δρ_max_ = 0.29 e Å^−^^3^
0 restraints	Δρ_min_ = −0.50 e Å^−^^3^

### Special details

**Table:**

Geometry. All e.s.d.'s (except the e.s.d. in the dihedral angle between two l.s. planes) are estimated using the full covariance matrix. The cell e.s.d.'s are taken into account individually in the estimation of e.s.d.'s in distances, angles and torsion angles; correlations between e.s.d.'s in cell parameters are only used when they are defined by crystal symmetry. An approximate (isotropic) treatment of cell e.s.d.'s is used for estimating e.s.d.'s involving l.s. planes.
Refinement. Refinement of *F*^2^ against ALL reflections. The weighted *R*-factor *wR* and goodness of fit *S* are based on *F*^2^, conventional *R*-factors *R* are based on *F*, with *F* set to zero for negative *F*^2^. The threshold expression of *F*^2^ > σ(*F*^2^) is used only for calculating *R*-factors(gt) *etc*. and is not relevant to the choice of reflections for refinement. *R*-factors based on *F*^2^ are statistically about twice as large as those based on *F*, and *R*- factors based on ALL data will be even larger.

### Fractional atomic coordinates and isotropic or equivalent isotropic displacement parameters (Å^2^)

**Table:**

	*x*	*y*	*z*	*U*_iso_*/*U*_eq_	
S1	0.38152 (6)	0.42510 (2)	0.02208 (3)	0.02374 (11)	
S2	0.98715 (5)	0.412867 (18)	0.30849 (3)	0.01609 (10)	
S3	1.13698 (6)	0.28550 (2)	0.44362 (3)	0.02327 (11)	
N1	0.6131 (2)	0.34137 (6)	0.21795 (9)	0.0164 (2)	
N2	0.7444 (2)	0.30069 (6)	0.29841 (9)	0.0173 (2)	
C1	0.1243 (3)	0.43987 (9)	−0.06539 (13)	0.0309 (4)	
H1	0.0995	0.4737	−0.1215	0.037*	
C2	−0.0402 (3)	0.39739 (10)	−0.04503 (13)	0.0308 (4)	
H2	−0.1931	0.3985	−0.0855	0.037*	
C3	0.0401 (3)	0.35160 (8)	0.04254 (12)	0.0226 (3)	
H3	−0.0520	0.3185	0.0677	0.027*	
C4	0.2703 (2)	0.36066 (8)	0.08760 (11)	0.0176 (3)	
C5	0.4082 (2)	0.32207 (7)	0.17652 (11)	0.0171 (3)	
H5	0.3479	0.2825	0.2044	0.020*	
C6	0.6657 (3)	0.23282 (8)	0.32401 (13)	0.0230 (3)	
H6A	0.7881	0.2090	0.3753	0.035*	
H6B	0.6211	0.2057	0.2568	0.035*	
H6C	0.5352	0.2382	0.3571	0.035*	
C7	0.9478 (2)	0.32789 (7)	0.35022 (11)	0.0161 (3)	
C8	1.2618 (2)	0.43443 (8)	0.39547 (11)	0.0182 (3)	
H8A	1.2569	0.4301	0.4733	0.022*	
H8B	1.3797	0.4028	0.3809	0.022*	
C9	1.3146 (2)	0.50761 (8)	0.36994 (11)	0.0167 (3)	
C10	1.4672 (2)	0.52084 (8)	0.30438 (11)	0.0186 (3)	
H10	1.5329	0.4834	0.2737	0.022*	
C11	1.5239 (3)	0.58827 (8)	0.28352 (12)	0.0212 (3)	
H11	1.6290	0.5967	0.2393	0.025*	
C12	1.4274 (3)	0.64340 (8)	0.32713 (12)	0.0217 (3)	
H12	1.4663	0.6894	0.3129	0.026*	
C13	1.2733 (3)	0.63085 (8)	0.39186 (12)	0.0217 (3)	
H13	1.2065	0.6684	0.4217	0.026*	
C14	1.2175 (2)	0.56349 (8)	0.41276 (11)	0.0204 (3)	
H14	1.1120	0.5553	0.4568	0.025*	

### Atomic displacement parameters (Å^2^)

**Table:**

	*U*^11^	*U*^22^	*U*^33^	*U*^12^	*U*^13^	*U*^23^
S1	0.0266 (2)	0.0239 (2)	0.01970 (19)	0.00074 (14)	0.00326 (14)	0.00408 (13)
S2	0.01503 (17)	0.01798 (18)	0.01359 (17)	−0.00058 (12)	−0.00020 (12)	0.00121 (11)
S3	0.02006 (18)	0.0231 (2)	0.0235 (2)	0.00380 (13)	−0.00161 (14)	0.00613 (14)
N1	0.0182 (6)	0.0174 (6)	0.0126 (5)	0.0008 (4)	0.0012 (4)	0.0017 (4)
N2	0.0183 (6)	0.0169 (6)	0.0151 (5)	−0.0003 (5)	0.0005 (4)	0.0031 (4)
C1	0.0414 (10)	0.0315 (9)	0.0169 (7)	0.0162 (7)	0.0005 (7)	−0.0005 (6)
C2	0.0237 (8)	0.0413 (10)	0.0227 (8)	0.0135 (7)	−0.0043 (6)	−0.0136 (7)
C3	0.0194 (7)	0.0290 (8)	0.0188 (7)	−0.0013 (6)	0.0030 (5)	−0.0106 (6)
C4	0.0180 (6)	0.0198 (7)	0.0145 (6)	−0.0010 (5)	0.0028 (5)	−0.0048 (5)
C5	0.0180 (6)	0.0176 (6)	0.0157 (6)	−0.0028 (5)	0.0041 (5)	−0.0016 (5)
C6	0.0237 (7)	0.0184 (7)	0.0254 (7)	−0.0028 (6)	0.0025 (6)	0.0057 (6)
C7	0.0160 (6)	0.0190 (6)	0.0139 (6)	0.0016 (5)	0.0044 (5)	−0.0009 (5)
C8	0.0153 (6)	0.0211 (7)	0.0162 (6)	0.0000 (5)	−0.0008 (5)	0.0001 (5)
C9	0.0136 (6)	0.0208 (7)	0.0130 (6)	0.0006 (5)	−0.0023 (5)	−0.0002 (5)
C10	0.0168 (6)	0.0221 (7)	0.0161 (6)	0.0031 (5)	0.0023 (5)	−0.0032 (5)
C11	0.0201 (7)	0.0270 (8)	0.0171 (7)	−0.0013 (6)	0.0056 (5)	0.0003 (6)
C12	0.0229 (7)	0.0215 (7)	0.0186 (7)	0.0000 (6)	0.0007 (6)	0.0014 (6)
C13	0.0234 (7)	0.0217 (7)	0.0194 (7)	0.0051 (6)	0.0038 (6)	−0.0023 (5)
C14	0.0191 (7)	0.0267 (8)	0.0161 (6)	0.0026 (6)	0.0055 (5)	−0.0007 (5)

### Geometric parameters (Å, º)

**Table:**

S1—C1	1.7139 (17)	C6—H6A	0.9800
S1—C4	1.7164 (15)	C6—H6B	0.9800
S2—C7	1.7610 (15)	C6—H6C	0.9800
S2—C8	1.8188 (14)	C8—C9	1.504 (2)
S3—C7	1.6594 (14)	C8—H8A	0.9900
N1—C5	1.2876 (18)	C8—H8B	0.9900
N1—N2	1.3821 (16)	C9—C10	1.3960 (19)
N2—C7	1.3615 (18)	C9—C14	1.398 (2)
N2—C6	1.4597 (18)	C10—C11	1.391 (2)
C1—C2	1.362 (3)	C10—H10	0.9500
C1—H1	0.9500	C11—C12	1.389 (2)
C2—C3	1.412 (2)	C11—H11	0.9500
C2—H2	0.9500	C12—C13	1.393 (2)
C3—C4	1.3915 (19)	C12—H12	0.9500
C3—H3	0.9500	C13—C14	1.388 (2)
C4—C5	1.443 (2)	C13—H13	0.9500
C5—H5	0.9500	C14—H14	0.9500
			
C1—S1—C4	91.75 (8)	N2—C7—S3	123.41 (11)
C7—S2—C8	101.72 (7)	N2—C7—S2	112.87 (10)
C5—N1—N2	118.12 (12)	S3—C7—S2	123.72 (8)
C7—N2—N1	115.86 (11)	C9—C8—S2	107.34 (9)
C7—N2—C6	123.27 (12)	C9—C8—H8A	110.2
N1—N2—C6	120.86 (11)	S2—C8—H8A	110.2
C2—C1—S1	112.07 (13)	C9—C8—H8B	110.2
C2—C1—H1	124.0	S2—C8—H8B	110.2
S1—C1—H1	124.0	H8A—C8—H8B	108.5
C1—C2—C3	112.96 (14)	C10—C9—C14	118.61 (14)
C1—C2—H2	123.5	C10—C9—C8	120.07 (13)
C3—C2—H2	123.5	C14—C9—C8	121.30 (13)
C4—C3—C2	111.92 (15)	C11—C10—C9	120.62 (13)
C4—C3—H3	124.0	C11—C10—H10	119.7
C2—C3—H3	124.0	C9—C10—H10	119.7
C3—C4—C5	126.83 (14)	C12—C11—C10	120.22 (14)
C3—C4—S1	111.30 (11)	C12—C11—H11	119.9
C5—C4—S1	121.87 (11)	C10—C11—H11	119.9
N1—C5—C4	119.83 (13)	C11—C12—C13	119.67 (14)
N1—C5—H5	120.1	C11—C12—H12	120.2
C4—C5—H5	120.1	C13—C12—H12	120.2
N2—C6—H6A	109.5	C14—C13—C12	119.97 (14)
N2—C6—H6B	109.5	C14—C13—H13	120.0
H6A—C6—H6B	109.5	C12—C13—H13	120.0
N2—C6—H6C	109.5	C13—C14—C9	120.90 (13)
H6A—C6—H6C	109.5	C13—C14—H14	119.6
H6B—C6—H6C	109.5	C9—C14—H14	119.6
			
C5—N1—N2—C7	−171.17 (12)	C6—N2—C7—S2	−175.83 (11)
C5—N1—N2—C6	9.13 (19)	C8—S2—C7—N2	177.56 (10)
C4—S1—C1—C2	0.23 (13)	C8—S2—C7—S3	−1.90 (10)
S1—C1—C2—C3	−0.11 (18)	C7—S2—C8—C9	−178.59 (9)
C1—C2—C3—C4	−0.10 (19)	S2—C8—C9—C10	−102.29 (13)
C2—C3—C4—C5	−178.82 (13)	S2—C8—C9—C14	79.22 (14)
C2—C3—C4—S1	0.27 (16)	C14—C9—C10—C11	0.9 (2)
C1—S1—C4—C3	−0.28 (12)	C8—C9—C10—C11	−177.58 (13)
C1—S1—C4—C5	178.86 (12)	C9—C10—C11—C12	−0.5 (2)
N2—N1—C5—C4	−176.07 (12)	C10—C11—C12—C13	0.0 (2)
C3—C4—C5—N1	−172.02 (13)	C11—C12—C13—C14	0.2 (2)
S1—C4—C5—N1	8.98 (19)	C12—C13—C14—C9	0.2 (2)
N1—N2—C7—S3	−176.06 (9)	C10—C9—C14—C13	−0.8 (2)
C6—N2—C7—S3	3.63 (19)	C8—C9—C14—C13	177.73 (13)
N1—N2—C7—S2	4.48 (15)		

### Hydrogen-bond geometry (Å, º)

Cg1 and Cg2 are the centroids of the S1,C1–C4 and C9–C14 rings, respectively.

**Table:**

*D*—H···*A*	*D*—H	H···*A*	*D*···*A*	*D*—H···*A*
C6—H6*A*···*Cg*1^i^	0.98	2.85	3.4021 (17)	117
C8—H8*A*···*Cg*2^ii^	0.99	2.80	3.4795 (15)	127

Symmetry codes: (i) *x*+1/2, −*y*+1/2, *z*+1/2; (ii) −*x*+3, −*y*+1, −*z*+1.
